# Role of CD19 and specific KIT‐D816 on risk stratification refinement in t(8;21) acute myeloid leukemia induced with different cytarabine intensities

**DOI:** 10.1002/cam4.3705

**Published:** 2020-12-31

**Authors:** Biao Wang, Bin Yang, Yun Ling, Jihong Zhang, Xiaoying Hua, Weiying Gu, Feng Yan

**Affiliations:** ^1^ Department of Hematology Changzhou First People's Hospital Changzhou China; ^2^ Blood Research Laboratory Shengjing Hospital of China Medical University Shenyang China

**Keywords:** acute myeloid leukemia, CD19, cytarabine, *KIT*, t(8;21)

## Abstract

High‐dose cytarabine (Ara‐C) has been reported with increased treatment‐related mortality, whereas few data are available concerning intermediate‐dose Ara‐C for induction of acute myeloid leukemia (AML) with t(8;21) translocation. We retrospectively analyzed factors impacting complete remission (CR), event‐free survival (EFS), cumulative incidence of relapse (CIR), and overall survival (OS) in 197 adults with t(8;21) AML, of whom 107 cases were induced with intermediate‐dose and 90 with standard‐dose Ara‐C (as part of 3 + 7 protocol). After a single induction course, the overall CR rate was 87.6% (170/194), with a significant difference between the standard‐dose (83/105, 79.0%) and intermediate‐dose (87/89, 97.8%) groups (*p* < 0.001). Rather than general *KIT*mut, the specific *KIT*‐D816 independently led to a lower probability of achieving CR (*HR* = 3.29 [1.18–9.24], *p* = 0.023), worse EFS (*HR* = 3.53 [1.82–6.84], *p* < 0.001), and OS (*HR* = 5.45 [1.77–16.84], *p* = 0.003) in the standard‐dose group, but not in the intermediate‐dose group. CD19(+) represented the only independent factor predicting lower CIR both in the standard‐dose group (*HR* = 0.32 [0.10–1.00], *p* = 0.050) and in the intermediate‐dose group (*HR* = 0.11 [0.03–0.40], *p* = 0.001). When combined, *KIT*(+) plus CD19(−) conferred the most increased relapse risk (3‐year CIR 60%; SE 0.12). Specific *KIT*‐D816, instead of general *KIT*mut, may be incorporated in prognostication model for t(8;21) AML. Combination of CD19 with *KIT* provides a more definite risk stratification profile for t(8;21) AML.

## Graphical abstract

We refined heterogeneous factors predicting clinical outcome in t(8;21) AML patients under induction with different cytarabine intensities.The specific KIT‐D816 (rather than general KITmut) is more associated with response, EFS, and OS under standard‐dose Ara‐C induction, and induction chemotherapy containing intermediate‐dose Ara‐C abrogates response disadvantage caused by KIT‐D816.Combination of CD19 and KIT can deliver a more explicit risk stratification profile and elaborately guide an individually risk‐adapted treatment strategy in t(8;21) AML.

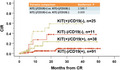

## 1 INTRODUCTION

Acute myeloid leukemia (AML) with t(8;21)(q22;q22.1)/*RUNX1*‐*RUNX1 T1* is categorized as an individual disease entity in the World Health Organization (WHO) classification of myeloid neoplasms and acute leukemia.^1^ Compared with other cytogenetic subtypes, t(8;21) AML has relatively superior survival. However, there is still considerable clinical heterogeneity in this AML subtype, as indicated by a relapse rate up to 40% and probability of overall survival (OS) between 40% and 60%.^2^, ^3^, ^4^, ^5^, ^6^

Most of the previous reports focused on the effect of cytogenetics on clinical outcomes of t(8;21) AML. The prognostic significance of commonly occurring additional cytogenetic aberrations has been described with inconsistent or even inverse conclusions. For instance, loss of a sex chromosome,^3^, ^7^ additional del(9q),^8^, ^9^ additional +4,^10^, ^11^ and additional three or more chromosomal abnormalities^12^, ^13^ have been variedly reported.

Emerging investigations have identified large numbers of significative mutations potentially holding leukemogenic interplay with oncofusion proteins. *KIT* mutations (*KIT*mut) had been the most largely studied, but its prognostic significance has been variedly described with somewhat controversial conclusions. The latest National Comprehensive Cancer Network (NCCN) guidelines^14^ categorized t(8;21) AML with *KIT*mut as an intermediate risk,^14^ while the 2017 European LeukemiaNet (ELN) Recommendations classified t(8;21) AML as a favorable risk regardless of *KIT* mutational status.^15^

Immunophenotypically, t(8;21) AML has been demonstrated as exhibiting unique antigenic features, which is characterized by aberrant expression of cross‐lineage CD19 and CD56.^16^, ^17^ The B‐cell marker CD19 in t(8;21) AML is also associated with negativity for *KIT* mutation,^18^ and shows a prognostic relevance with relapse even in patient subsets without the *KIT* exon 17 mutation.^9^, ^19^, ^20^ However, molecular genetic information was not or less integrated into analyses of these studies.

Repeated cycles of high‐dose cytarabine (Ara‐C) as post‐remission treatment can improve prognosis in t(8;21) AML.^21^ We wonder whether a dosage of Ara‐C above standard‐dose level in induction may benefit clinical outcome for this AML subtype. According to a review regarding dose–efficacy relationship, a level of intermediate‐dose Ara‐C in each treatment cycle in AML is enough, while high‐dose Ara‐C seems to make no sense.^22^ Since high‐dose Ara‐C may increase the risk of induced toxicity,^23^ we did not consider the use of high‐dose Ara‐C to induce AML in our regimen design. Most of the earlier studies did not comprehensively integrate the genomics‐based prognostication scheme, resultantly the conclusions could not reflect the concept of risk‐adapted therapy using high‐dose Ara‐C.

In this study, we performed a retrospective analysis to evaluate the clinical outcomes of patients with t(8;21) AML integrating the traditional clinicopathological and genomic features, as well as including standard‐dose Ara‐C 100–200 mg/m^2^ and intermediate‐dose Ara‐C 1000–2000 mg/m^2^ induction as covariates. We aimed to optimize treatment options in the context of induction modalities containing different intensities of Ara‐C from this relatively favorable AML subtype.

## 2 PATIENTS AND METHODS

### 2.1 Study population

**FIGURE 1:**
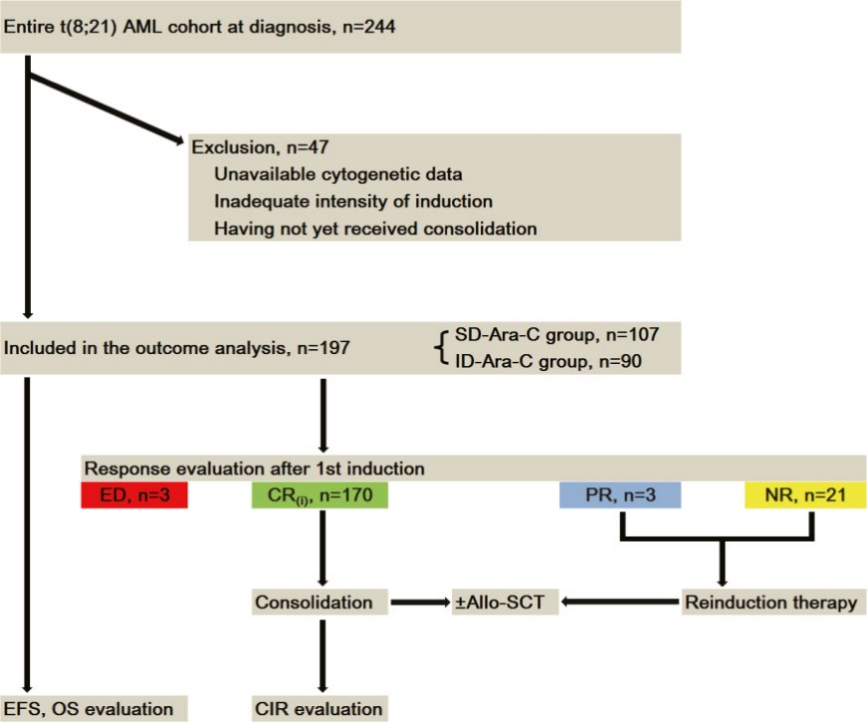
Flowchart of t(8;21) AML patient selection for outcome analysis. Ultimately, available data on the final cohort of 197 patients are included in the response and survival evaluation; AML, acute myeloid leukemia; SD‐Ara‐C, standard‐dose Ara‐C; ID‐Ara‐C, intermediate‐dose Ara‐C; ED, early death; CR, complete remission; PR, partial remission; NR, no remission; Allo‐SCT, allogeneic stem cell transplantation; EFS, event‐free survival; OS, overall survival; CIR, cumulative incidence of relapse

A total of 244 newly diagnosed patients with t(8;21) AML from our institute and Shengjing Hospital of China Medical University from August 2014 to March 2019 were included in this study. Among them, 197 cases were eligible for this study due to the availability of cytogenetic data and had completed at least one course of post‐remission consolidation for those having reached a CR. A flow chart for the study cohort of t(8;21) AML patients is shown in Figure 1.

### 2.2 Clinicopathological work‐ups

Patients' routine MICM work‐ups were carried out and diagnosis was established according to the 2008 WHO criteria.^24^ Bone marrow aspirates from t(8;21) AML patients admitted at our and cooperating hospitals were analyzed by the 8‐color multiparameter flow cytometry for a leukemia‐associated phenotype, as described in our previous study.^25^ The presence of t(8;21) translocations was mostly determined by conventional karyotyping, and confirmed by fluorescence in situ hybridization and/or reverse transcription polymerase chain reaction detecting *RUNX1*‐*RUNX1 T1* fusion genes or transcripts. This study was conducted in accordance with the Declaration of Helsinki and the protocol was approved by the Institutional Review Boards of all participating institutions. All patients provided written informed consent for receiving therapies and using their records.

### 2.3 Next‐generation sequencing (NGS)

Massively parallel sequencing was performed on MiSeq/HiSeq (Illumina) or Ion torrent PGM™ (Life Technologies) platforms. A custom‐designed panel of oligonucleotide probe was made to capture the exons of 112 potentially mutated genes involved in hematological diseases as previously reported.^25^ Sequencing reads in FASTQ format were aligned to the human reference genome (GRCh38) using Burrows‐Wheeler Aligner (BWA, v0.6) and SAMtools algorithm. Variant callings for somatic alterations, including single nucleotide variants (SNVs) and short fragment indels in protein coding sequence (CDS), were analyzed using multiple pipelines (Ion Reporter™ and Variant Reporter) and annotated referencing to the dbSNP (Single Nucleotide Polymorphism database), 1000 Genomes, PolyPhen‐2, and COSMIC (Catalogue Of Somatic Mutations In Cancer) databases. Additionally, the PCR followed by direct sequencing was also performed to detect *FLT3*‐ITD, *NPM1*, and *CEBPA* for their potential complex indels as previously described.^26^, ^27^, ^28^

### 2.4 Induction and consolidation treatment

Patients eligible for response and outcome analyses were induced with either standard‐ or intermediate‐dose Ara‐C containing regimen as follows: (i) standard‐dose Ara‐C group consisted of Ara‐C 100–200 mg/m^2^ given by continuous IV over 24 h on days 1–7, plus daunorubicin 60 mg/m^2^ or idarubicin 12 mg/m^2^ rapid IV on days 1–3; (ii) intermediate‐dose Ara‐C group consisted of Ara‐C 1000–2000 mg/m^2^ IV over 2 h per 12 h on days 5–7, with Ara‐C on days 1–4 and daunorubicin/idarubicin at the same dose and schedule as standard‐dose group. BM response assessment was performed between day 21 and day 28 after induction when peripheral blood (PB) counts recovered. When no PB recovery was noticed or leukemic blasts persisted or reappeared in PB, the response assessment was postponed not later than day 35 after induction. Patients obtaining a complete remission (CR) or CR with incomplete hematologic recovery (CRi) received consolidation chemotherapies based on: (i) high‐dose single‐agent Ara‐C 3000 mg/m^2^ given by 3‐hour IV per 12 h on days 1, 3, and 5; or (ii) intermediate‐dose Ara‐C 1500–2000 mg/m^2^ per 12 h for 4 days, with or without an anthracycline as above.

### 2.5 Clinical endpoints and definitions

The probability of achieving CR after a single course, cumulative incidence of relapse (CIR), event‐free survival (EFS), and OS was evaluated. The CIR was calculated from CR date to relapse date, with death from any cause in the absence of relapse as a competing event. Definitions of other endpoints (i.e., CR, CRi, EFS, and OS) are according to response criteria of the 2017 ELN Recommendations on AML.^15^ Because regimens containing higher doses of Ara‐C are generally considered as an optimal option for patients not responding to a first cycle of standard‐dose Ara‐C, the intensive intermediate‐dose Ara‐C induction is more likely to well identify primary resistance. To ensure the comparability between subgroups, in the present study, we evaluated the response after a single induction course instead of one or two courses. Patients who underwent allogeneic stem cell transplantation (allo‐SCT) were censored from survival analysis on the date of transplantation. Among patients who achieved a CR/CRi, only those who had received at least one cycle of post‐remission consolidation were included in the outcome analysis. This was a retrospective study without randomization to standard‐ versus intermediate‐dose induction.

### 2.6 Statistical analysis

Median values (range) were presented for non‐normally distributed continuous data, on which non‐parametric Mann‐Whitney U test was done for analysis. Frequencies were compared by Chi‐square test for categorical variables after cross‐tabulation. Continuous variables were dichotomously transformed when divided near their median values. Probabilities of survival outcome were estimated using the Kaplan‐Meier method, and compared by Log‐rank test between groups. Exceptionally, the CIR was compared using Fine and Gray model. Comparisons between groups according to clinicopathological parameters were also layered by induction intensity, and vice versa. Factors that fulfilled the prespecified assumption with *P*‐values <0.15 from univariate analyses were further included as covariates in multivariate logistic or Cox regression model. All statistical tests were two‐sided and *P*‐values < 0.05 were considered significant. Statistical analyses were performed using IBM SPSS Statistics 25 for Windows and Stata/SE 15.0.

## 3 RESULTS

### 3.1 Presenting clinicopathological and genetic features

The median age was 34 (range 16–71) years for the total 207 newly diagnosed t(8;21) patients, including 109 males and 98 females. On evaluable metaphases grounds, chromosomal t(8;21) was morphologically absent in 11 (5.3%) cases, of whom five had normal karyotypes and the remaining six had only additional abnormalities other than t(8;21). Of the 207 cases, complete NGS data records were available in 186 cases, in whom four cases (2.2%) were identified to carry none of the mutations. The demographic and baseline features of MICM and relatively commonly mutated genes (>5% of mutational incidence) at diagnosis are listed in Tables S1 and S2, respectively.

### 3.2 Bias test between induction groups

Prior to analyses of response and outcome, we tested the baseline balance across induction groups. More patients with age younger than 35 years (*p* = 0.020), hemoglobin level below 80 g/L (*p* = 0.017), and *KIT*‐D816 mutations (*p* = 0.005) were treated with intermediate‐dose Ara‐C during the induction (Tables S1 and S2). The distribution of other baseline parameters was parallel between standard‐ and intermediate‐dose groups. The consolidation cycles were evenly distributed between the two induction groups (mean 2.8 and 2.2 cycles, respectively; *p* = 0.593; data not shown). The distribution of patients undergoing allo‐SCT in first CR (CR1) was also balanced, with eight cases (8/105, 7.6%) in standard‐dose and five (5/89, 5.6%) in intermediate‐dose arm, respectively (*p* = 0.579; data not shown).

### 3.3 Role of intermediate‐dose induction and *KIT*‐D816 in affecting remission

Ten patients were excluded due to inadequate intensity of induction agent dosages. The remaining 197 patients who met the criteria were included in the response and survival analyses. Among them, 107 patients were induced with standard‐dose Ara‐C, and 90 with intermediate‐dose Ara‐C. Three cases (1.5%) had early death of infectious sepsis or cerebral hemorrhage, including two cases in standard‐dose and one in intermediate‐dose group, respectively. The remaining 194 cases were assessed for response. After a single induction course, the overall CR rate in entire cohort was 87.6% (170/194), with a significant difference between standard‐dose (83/105, 79.0%) and intermediate‐dose groups (87/89, 97.8%), respectively (*p* < 0.001). When the study cohort was layered by varying baselines as listed in Tables S1 and S2 at dichotomous levels, the intermediate‐dose Ara‐C could produce improvement on CR rate compared with standard‐dose Ara‐C within majority of the layers (data not shown).

Patients harboring *KIT*mut had lower CR rate than those without *KIT*mut in entire cohort (80.0% vs. 93.9%, *p* = 0.005), with difference remaining significant for *KIT*‐D816 (73.7% vs. 91.4%, *p* = 0.008) instead for *KIT*‐N822 (83.8% vs. 88.7%, *p* = 0.603, data not shown). This meant that the decrease of CR rate caused by *KIT*mut was predominantly attributable to D816 rather than other codon changes such as N822 (Table S3). Induction‐layered analysis showed that *KIT*mut and *KIT*‐D816 adversely affected CR rate in standard‐dose group (*p* = 0.029 and *p* = 0.020, respectively), instead of in intermediate‐dose group (*p* = 0.167 and *P* = 1.000, respectively), pointing to the benefit from intermediate‐dose Ara‐C to overcome the adverse induction response caused by *KIT*mut or *KIT*‐D816.

We further analyzed the factors predicting the probability of achieving CR using multivariate logistic regression analysis. Results indicated an independent association of standard‐dose Ara‐C induction (*HR* = 0.09 [0.02–0.40], *p* = 0.002) and *KIT*mut (*HR* = 3.58 [1.28–10.03], *p* = 0.015) with lower probability of achieving CR in entire cohort. It is noteworthy that the age and *KIT*‐D816 distribution were not balanced between both induction groups, with older and *KIT*‐D816 patients more frequently allocated in the standard‐dose group. Adjustment for age (either as a continuous or categorical variable) and *KIT*‐D816 to the covariates was then conducted in multivariate model within the entire cohort, yielding results that Ara‐C intensity remained as the powerful and robust predictor to induction response (*HR* = 0.09 [0.02–0.41], *p* = 0.002).

Induction‐layered analysis identified *KIT*‐D816 as having independent association with higher probability of achieving CR in the standard‐dose group (*HR* = 3.29 [1.18–9.24], *p* = 0.023), while none of the predictors was identified in the intermediate‐dose group (Table 1).

**TABLE 1:** Multivariate logistic analysis for the probability of attaining CR in entire t(8;21) cohort and in induction layers

Factors	Good	Entire cohort			SD Ara‐C			ID Ara‐C		
		*χ^2^ p*	HR (95% CI)	*p*	*χ^2^ p*	HR (95% CI)	*p*	*χ^2^ p*	HR (95% CI)	*p*
WBC count^a^	<10	***0.033***	NA	NA	0.058	NA	NA	0.220	NA	NA
CD19	(+)	0.136	NA	NA	0.453	NA	NA	1.000	NA	NA
*KIT*	(−)	***0.005***	***3.58 (1.28–10.03)***	***0.015***	***0.029***	NA	NA	0.167	NA	NA
*KIT*‐D816	(−)	***0.008***	NA	NA	***0.020***	***3.29 (1.18–9.24)***	***0.023***	1.000	NA	NA
Induction	ID	***<0.001***	***0.09 (0.02–0.40)***	***0.002***	NA	NA	NA	NA	NA	NA

Abbreviations and Annotations: SD, standard‐dose; ID, intermediate‐dose; Ara‐C, cytarabine; WBC, white blood cell; NA, not applicable.

^a^ tested as dichotomous variable divided near its median value. Multivariate logistic regression analysis was performed on forward selection method for factors fulfilling presupposed P<0.15 from univariate Chi‐square test in entire cohort and in both induction arms. Parameters showing statistical significance are highlighted in bold and italic.

### 3.4 Independent implication of CD19 for predicting relapse risk

Multivariate Cox model indicated sex (male), positive CD19, and wild‐type *NOTCH1* to be independently predictive of a lower CIR versus their counterparts in entire cohort (sex: *HR *= 0.28 [0.11–0.69], *p* = 0.006; CD19: *HR* = 0.117 [0.05–0.28], *p* < 0.001; *NOTCH1*: *HR* = 14.10 [3.57–55.72], *p* < 0.001, respectively).

**FIGURE 2:**
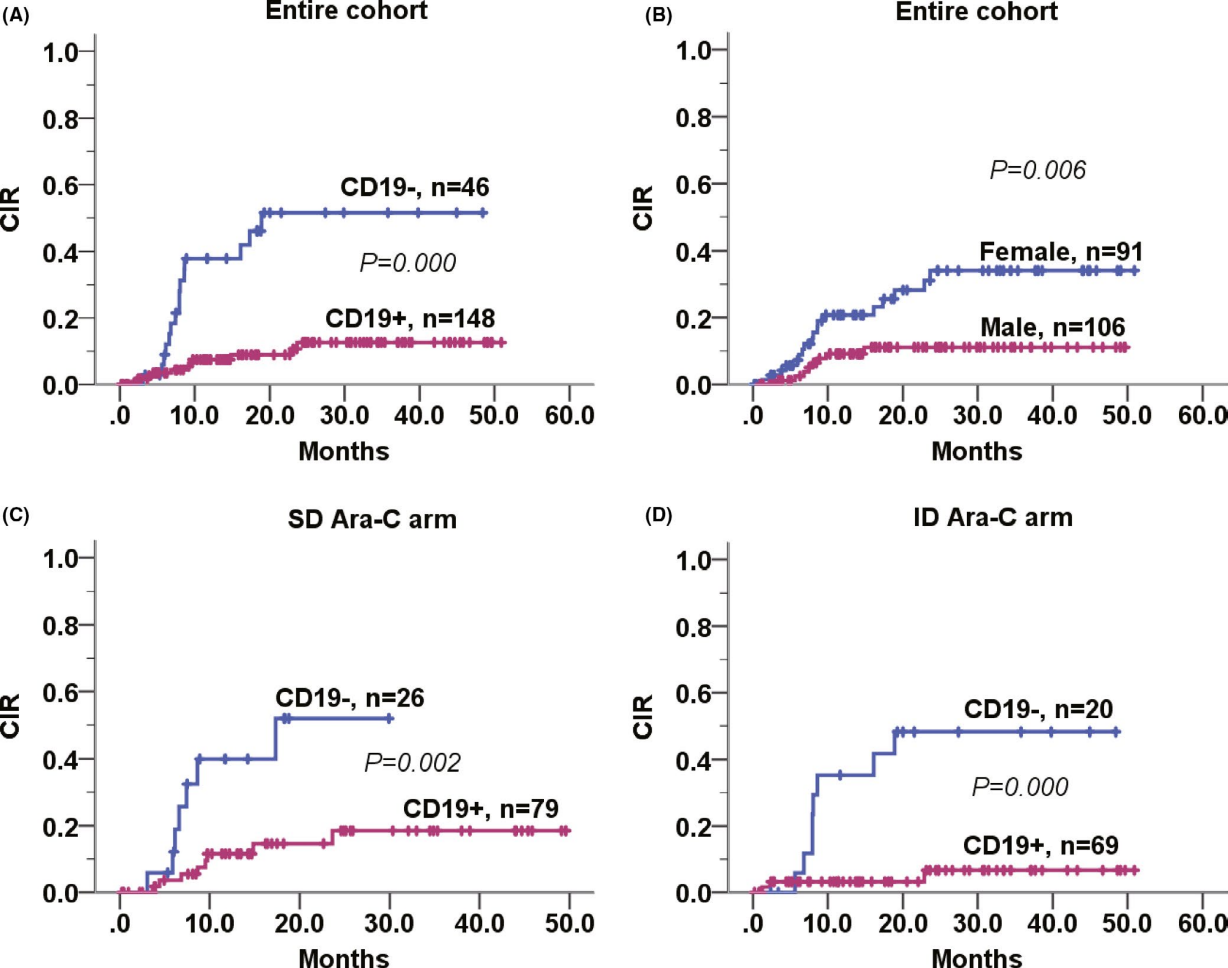
Estimated CIR according to CD19 or sex in entire t(8;21) cohort and in both induction layers. Patients with negative CD19 (A) or female sex (B) experienced more elevated CIR compared to their counterparts in entire t(8;21) cohort (*p* < 0.001 for CD19; *p* = 0.006 for sex, respectively). Induction‐layered outcome analysis revealed only CD19 as independently predicting relapse risk both in the standard‐ (C) and in the intermediate‐dose Ara‐C groups (D), as illustrated in Kaplan‐Meier curves by Log‐rank test (*p* = 0.002 and *p* < 0.001, respectively). CIR, cumulative incidence of relapse; SD, standard‐dose; ID, intermediate‐dose

Although *KIT*mut and *KIT*‐D816 also were associated with more higher CIR from univariate Kaplan‐Meier analysis, induction‐layered analysis identified CD19 as the only factor predicting CIR both in the standard‐dose group (*HR* = 0.32 [0.10–1.00], *p* = 0.050) and in the intermediate‐dose group (*HR* = 0.11 [0.03–0.40], *p* = 0.001) independently of *KIT* mutational status (Table 2 and Figure 2).

**TABLE 2:** Multivariate cox model of CIR in entire t(8;21) cohort and in induction layers

Factors	Good	Kaplan‐Meier on CIR			Multivariate Cox on CIR					
		*p* in Entire	*p* in SD	*p* in ID	HR (95% CI) in Entire	*p*	HR (95% CI) in SD	*p*	HR (95% CI) in ID	*p*
Sex	Male	***0.006***	0.079	***0.041***	***0.28 (0.11–0.69)***	***0.006***	NA	NA	NA	NA
CD19	(+)	***<0.001***	***0.002***	***<0.001***	***0.12 (0.05–0.28)***	***<0.001***	***0.32 (0.10–1.00)***	***0.050***	***0.11 (0.03–0.40)***	***0.001***
*KIT*	(−)	***0.001***	***0.015***	***0.020***	NA	NA	NA	NA	NA	NA
*KIT*‐D816	(−)	***<0.001***	***0.003***	***0.013***	NA	NA	NA	NA	NA	NA
*NRAS*	(+)	***0.027***	0.090	0.152	NA	NA	NA	NA	NA	NA
*NOTCH1*	(−)	***0.020***	0.228	***0.004***	***14.10 (3.57–55.72)***	***<0.001***	NA	NA	NA	NA

Abbreviations and Annotations: CIR, cumulative incidence of relapse; SD, standard‐dose; ID, intermediate‐dose; NA, not applicable; Parameters showing statistical significance are highlighted in bold and italic.

### 3.5 Correlation of specific *KIT*‐D816 or CD19 with EFS

In a multivariate Cox model for entire cohort, factors predictive of inferior EFS were sex (female) (*HR* = 0.54 [0.30–0.98], *p* = 0.042), negative CD19 (*HR* = 0.37 (0.20–0.70), *p* = 0.004), mutated *KIT* (*HR* = 3.01 (1.56–5.81), *p* = 0.001), *JAK2* (*HR* = 2.95 (1.27–6.83), *p* = 0.012), *NOTCH1* (*HR* = 3.74 [1.57–9.07], *p* = 0.003), *TET2* (*HR* = 2.75 [1.30–5.82], *p* = 0.008), and standard‐dose induction (*HR *= 0.33 [0.17–0.65], *p* = 0.001) (Table 3). Issue of unbalanced assignment for age and *KIT*‐D816 was needed to be taken into account across groups of different induction intensity, which per se also had clinical implication on EFS, while adjustment by them in multivariate Cox analysis did not alter the above findings.

**TABLE 3:** Multivariate cox model of EFS in entire t(8;21) cohort and in induction layers

Factors	Good	Kaplan‐Meier on EFS			Multivariate Cox on EFS					
		*p* in Entire	*p* in SD	*p* in ID	HR (95% CI) in Entire	*p*	HR (95% CI) in SD	*p*	HR (95% CI) in ID	*p*
Sex	Male	0.089	0.555	***0.031***	***0.54 (0.30–0.98)***	***0.042***	NA	NA	NA	NA
PLT	<30	0.217	***0.019***	0.589	NA	NA	NA	NA	NA	NA
CD19	(+)	***<0.001***	***0.003***	***<0.001***	***0.37 (0.20–0.70)***	***0.004***	NA	NA	***0.18 (0.06–0.54)***	***0.002***
CD79a	(+)	0.149	0.449	0.066	NA	NA	NA	NA	NA	NA
*KIT*	(−)	***<0.001***	***<0.001***	***0.005***	***3.01 (1.56–5.81)***	***0.001***	NA	NA	NA	NA
*KIT*‐D816	(−)	***<0.001***	***<0.001***	***0.040***	NA	NA	***3.53 (1.82–6.84)***	***<0.001***	NA	NA
*KIT*‐N822	(−)	0.081	0.071	0.298	NA	NA	NA	NA	NA	NA
*JAK2*	(−)	0.070	0.159	0.154	***2.95 (1.27–6.83)***	***0.012***	NA	NA	NA	NA
*NRAS*	(+)	0.116	0.067	0.974	NA	NA	NA	NA	NA	NA
*NOTCH1*	(−)	***0.005***	0.114	***0.042***	***3.74 (1.57–9.07)***	***0.003***	NA	NA	NA	NA
*TET2*	(−)	***0.037***	***0.044***	0.202	***2.75 (1.30–5.82)***	***0.008***	NA	NA	NA	NA
Induction	ID	***0.001***	NA	NA	***0.33 (0.17–0.65)***	***0.001***	NA	NA	NA	NA

Abbreviations and Annotations: EFS, event‐free survival; SD, standard‐dose; ID, intermediate‐dose; NA, not applicable; Parameters showing statistical significance are highlighted in bold and italic.

**FIGURE 3:**
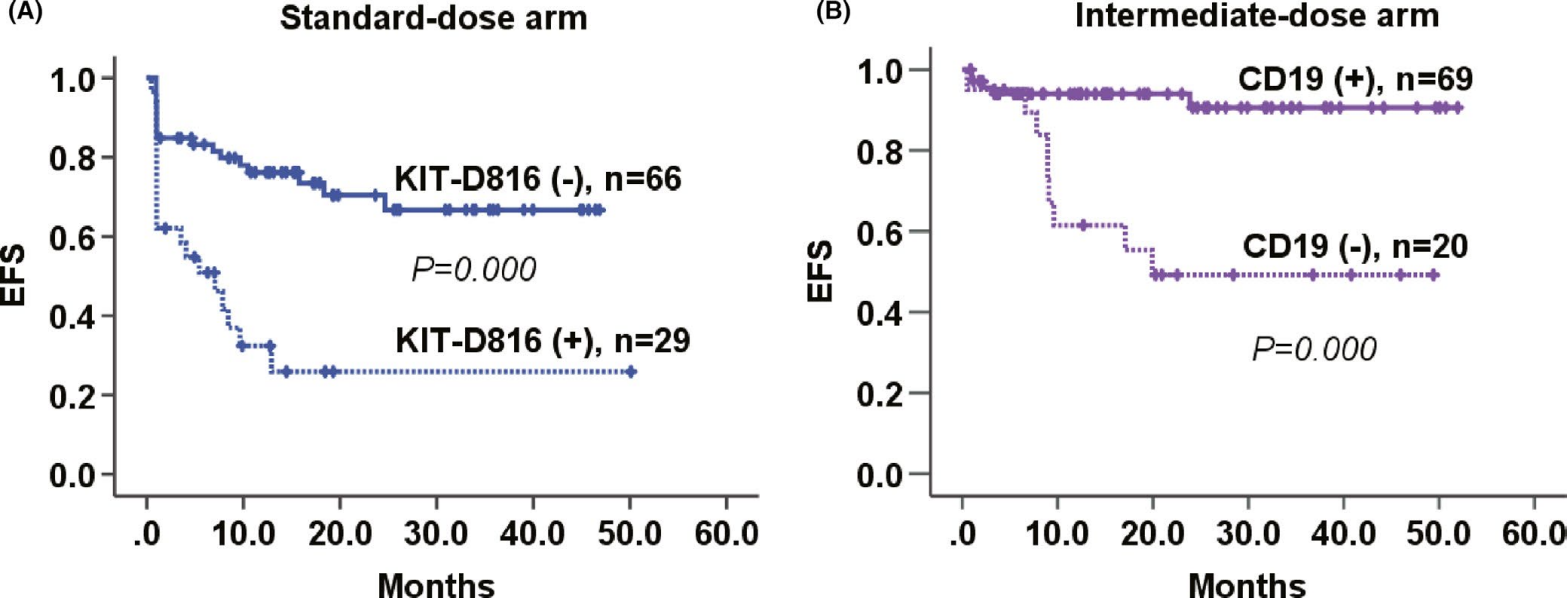
Estimated EFS according to *KIT*‐D816 in SD arm and CD19 in ID arm. (A) Induction‐layered multivariate Cox analysis showed that, in the standard‐dose Ara‐C group, only the specific *KIT*‐D816 was independently associated with inferior EFS, as illustrated in Kaplan‐Meier curve by Log‐rank test (*p* < 0.001). (B) In the intermediate‐dose Ara‐C group, positive CD19 was the only marker independently predictive of superior EFS, as illustrated in Kaplan‐Meier curve by Log‐rank test (*p* < 0.001). EFS, event‐free survival; SD, standard‐dose; ID, intermediate‐dose

Induction‐layered analysis showed only *KIT*‐D816 as an independent predictor for worse EFS in the standard‐dose group (*HR* = 3.53 [1.82–6.84], *p* < 0.001). Whereas in the intermediate‐dose group, positive CD19 was the only marker independently associated with superior EFS (*HR* = 0.18 [0.06–0.54], *p* = 0.002) (Table 3 and Figure 3). In view of the aforementioned results of CR rate and CIR, the *KIT*‐D816 adversely affected EFS mainly by decreasing the probability of achieving CR, whereas CD19 mainly by affecting CIR.

Additionally, mutated *NRAS* showed a tendency toward superior EFS from univariate analysis (*p* = 0.067) in the standard‐dose group, mostly by reducing CIR (*p* = 0.090), but it had no independent significance in multivariate Cox analysis.

### 3.6 Correlation of specific *KIT*‐D816 or CD19 with OS

According to a Cox model, *KIT*mut independently resulted in worse OS (*HR* = 4.18 [1.49–11.74], *p* = 0.007) in entire cohort when censoring survival analysis for allotransplanted patients at date of transplantation. Once again, *KIT*‐D816 was identified as the only factor predicting worse OS in the standard‐dose group (*HR* = 5.45 [1.77–16.84], *p* = 0.003), and only CD19 in the intermediate‐dose group (*HR* = 0.16 [0.03–0.89], *p* = 0.037) (Table 4).

**TABLE 4:** Multivariate cox model of OS in entire t(8;21) cohort and in induction layers

Factors	Good	Kaplan‐Meier on OS				Multivariate Cox on OS					
		*p* in Entire	*p* in SD	*p* in ID		HR (95% CI) in Entire	*p*	HR (95% CI) in SD	*p*	HR (95% CI) in ID	*p*
CD19	(+)	***0.011***	0.128	***0.017***		NA	NA	NA	NA	***0.16 (0.03–0.89)***	***0.037***
AKAs	<3	***0.028***	***0.039***	0.689		NA	NA	NA	NA	NA	NA
*KIT*	(−)	***0.003***	***0.013***	0.211		***4.18 (1.49–11.74)***	***0.007***	NA	NA	NA	NA
*KIT*‐D816	(−)	***0.003***	***0.001***	0.339		NA	NA	***5.45 (1.77–16.84)***	***0.003***	NA	NA
*JAK2*	(−)	0.101	0.257	0.194		NA	NA	NA	NA	NA	NA
Induction	ID	0.051	NA	NA		NA	NA	NA	NA	NA	NA

Annotations and Abbreviations: OS, overall survival; SD, standard‐dose; ID, intermediate‐dose; NA, not applicable; AKAs, additional karyotypic abnormalities; Parameters showing statistical significance are highlighted in bold and italic.

Additionally, univariate analysis showed that extra three or more chromosomal abnormalities were associated with worse OS in entire cohort (*p* = 0.028) and in the standard‐dose group (*p* = 0.039), but it failed to reach a significance in multivariate Cox analysis.

### 3.7 Refinement of relapse risk stratification by combining *KIT* and CD19

**FIGURE 4:**
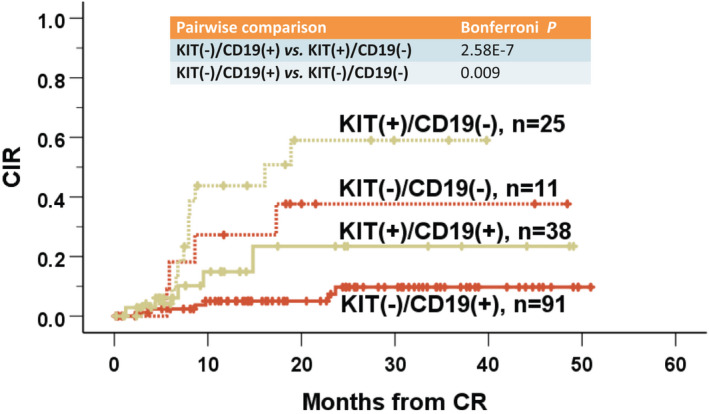
CIR according to *KIT* combined with CD19 in entire t(8;21) cohort. *KIT* mutational status in combination with CD19 expression conferred a more explicit risk strata for relapse in t(8;21) AML, with *KIT*(−)/CD19(+) cases experiencing the lowest CIR while *KIT*(+)/CD19(−) cases the highest CIR (3‐year CIR: 10% vs. 60%, respectively; Bonferroni‐adjusted *P* = 2.58E‐7). CIR, cumulative incidence of relapse

Since *KIT* and CD19 both presented independent relevance with relapse risk in multivariate Cox analysis, we subsequently performed a comparison in terms of their combination to estimate CIR. Given that they were implicated in both Ara‐C groups, and induction intensity per se had no impact on CIR, the comparison was conducted in entire cohort. Results demonstrated an even more explicit stratification aspect for prediction of relapse. Cases with *KIT*(−) plus CD19(+) showed the lowest relapse risk (3‐year CIR, 0.10; SE, 0.04), contrarily, *KIT*(+) plus CD19(−) showed the most increased relapse risk (3‐year CIR, 0.60; SE, 0.12), with a strikingly significant difference between the two subgroups (Bonferroni‐adjusted *P* = 2.58E‐7) (Figure 4).

## 4 DISCUSSION

Although t(8;21) AML is a favorable risk cytogenetic subtype, its clinical outcomes still harbor heterogeneities across individual patients. Concomitant *KIT*mut as prognostic factor still remains controversial for t(8;21) AML under current therapy modalities, as suggested by a subtle difference in the risk stratification between the 2017 ELN recommendations^15^ and the latest NCCN guidelines on AML.^14^ Moreover, although D816 represented the most common *KIT*mut type (45% of composition ratio), N822 and the remaining codons occupied the greater part in our cohort. Most of the previous reports have described adverse effect of *KIT*mut on prognosis in t(8;21) AML, but specific site or type of mutation has rarely been designated.

We here observed an unfavorable association of *KIT*mut with CR achievement in t(8;21) patients. For the two major *KIT*mut sites, codon D816 was mainly accountable for the response lesion compared with N822 and others, supported by in vitro studies demonstrating D816 to result in more stronger autonomous *KIT* phosphorylation and potent oncogenic transformation.^29^, ^30^

Standard‐dose Ara‐C combined with anthracyclines, that is, "7 + 3" scheme, has still exerted their cornerstone roles in modern AML therapy. Because anthracycline dosages in AML have reached their plateau (DNR 60–90 mg/m^2^ or IDA 12 mg/m^2^), the improvement of induction response of these "7 + 3" protocols can only place hope on dose adjustment of Ara‐C. The literature of high‐dose (HD) Ara‐C (2000–3000 mg/m^2^, every 12 h, for 3 days or more) used for AML chemotherapy was first published in 1985; afterwards, several randomized trials had been conducted and majorities failed to draw a conclusion of significant efficacy improvement as reported between 1991 and 2011, as reviewed by Lowenberg B.(22) However, prolonged follow‐up duration has later identified subsets of patients benefiting from high‐dose Ara‐C, such as AML with t(8;21)^31^ and Ras‐pathway mutations.^32^

Induction‐layered analysis indicated a decrease in CR rate caused by *KIT*mut (especially D816) as against wild‐type *KIT* under the standard‐dose Ara‐C induction. In contrast, the association between *KIT*mut with CR rate was inapparent when induced with intermediate‐dose Ara‐C, pointing to the inability of standard‐dose to overcome chemoresistance caused by *KIT*mut and that intermediate‐dose Ara‐C induction can be considered to improve induction response in this situation. Our result indicated significant improvements of CR rate and EFS from initial induction based on intermediate‐dose Ara‐C in t(8;21) patients, especially in those carrying *KIT*mut (distinctly D816). Similarly, a report on children with t(8;21) AML has shown that the cumulative dosage of Ara‐C over 3 g/m^2^ during the first induction course could significantly improve CR rate and EFS when compared to conventional dosage.^10^

Sequencing results are often not quickly available in clinical practice, which raises an issue of how to obtain the information of *KIT* status as soon as possible. Recently, detection of *KIT*mut by droplet digital PCR (ddPCR) technology has been developed.^33^ The presence of *KIT*‐D816, even with its low variant allele frequency (VAF), can potentially be quickly screened by this methodology. Furthermore, our usage of intermediate‐dose Ara‐C at the late stage (on day 5–7) of regimen schedule gives us ample opportunity to make appropriate dosage adjustment for this patient subsets. In addition, there have been tyrosine kinase inhibitors (TKIs) which can pharmacologically target *KIT*mut,^34^, ^35^, ^36^ but further clinical verification is needed.

As for prognostic significance of *KIT*mut, a large series of studies have described its inferior impact on outcome in t(8;21) AML. However, there was no clear conclusion in a recent meta‐analysis.^37^ We showed the predictive value of general *KIT*mut for inferior CIR, EFS, and OS in entire t(8;21) cohort from univariate analysis, while it lost independence for any of these endpoints in either induction group. The underrepresentation of *KIT*mut on outcome potentially may be attributable to the following reasons in that: (i) only the specific *KIT*‐D816 was more representative of causative adverse factor than N822, similar to other findings.^7^, ^38^ Furthermore, stratified analysis identified only *KIT*‐D816 as the main predictor in standard‐dose group. (ii) Repeated cycles of post‐remission high‐dose Ara‐C consolidation could still exert a role to continuously eliminate *KIT*mut clones and maintain survival benefit.^21^ (iii) By NGS, the *KIT*mut incidence in our cohort of t(8;21) patients was detected in 45.7%, a result relatively higher than other reports. Compared with traditional Sanger sequencing, the comparatively sensitive high‐throughput sequencing allowed us to detect *KIT*mut with lower VAF in a considerable proportion of patients. One report has indicated an association of elevated CIR with *KIT*mut only for cases with mutant level of 25% or higher.^39^ Unfortunately, we did not perform an analysis about this relationship. (iv) There existed diversification of clonal architecture as exemplified by late evolutionary signaling clones in the development and progression of AML. Besides *KIT*mut, mutations in *FLT3* and Ras pathways were also common in t(8;21) AML.^39^, ^40^, ^41^, ^42^ Very recently, one study described multiple signal clones of *KIT*, *FLT3*, and *NRAS*/*KRAS* variants as clonal interference,^43^ which conveyed an adverse EFS, while the presence of a single signaling clone did not affect prognosis. However, we showed mutated *NRAS* as presenting a non‐significantly favorable EFS and CIR inclination in standard‐dose group from our univariate analysis, congruent with a Germany multicenter children^10^ and French adults study.^44^

Determination of gene mutations was mainly based on traditional Sanger sequencing in earlier studies. With the enlargement and improvement of comprehensive sequencing spectrum and coverage in modern NGS platforms, more growing genetic lesions of potentially clinical relevance on varying loci within the *KIT* gene would be identified, as well as more coexisting genomic lesions, thereby giving profound insight into AML classification and informing prognostic stratification.^45^ The incidence of mutated *NOTCH1* (13/186, 7.0%), *JAK2* (16/186, 8.6%), and *TET2* (17/186, 9.1%), which were scarcely reported for their clinical implications in t(8;21) AML, was detected to be relatively lower but not rare in our cohort. We herein observed for the first time that *NOTCH1* and *JAK2* mutations served as adverse markers affecting outcome (*NOTCH1* for EFS and CIR; *JAK2* for EFS) in t(8;21) AML. *NOTCH1* mutations frequently occur in above 50% of T‐cell acute lymphoblastic leukemia cases.^46^ Activated *Notch* signaling contributes to the crosstalk between leukemia cells and surrounding mesenchymal stromal cells, leading to malignant property and chemoresistance.^47^, ^48^ We also observed an association of mutated *TET2* with inferior EFS, in line with Cher CY *et al* reporting that *TET2* mutations were an adverse prognostic factor in core binding factor AML.^49^ Further attention and clarification of clinical relevance would be required for mutated *NOTCH1*, *JAK2*, and *TET2* in t(8;21) AML.

There have been several studies concerning the effect of immunophenotyping on clinical outcome of t(8;21) AML. In contrast with a finding that CD19 was associated with CR rate with a borderline significance (expression group 95.7% vs. non‐expression group 83.8%; *p* = 0.049),^19^ we failed to reproduce a similar result. In our study of t(8;21) cohort, although intensified Ara‐C during induction could overcome chemoresistance resulting from *KIT*mut, it failed to prohibit higher risk of relapse resulting from CD19(−). Consistent with a previous study,^9^ a correlation was observed between CD19(−) and higher relapse risk in t(8;21) patients according to a Cox model. A more recent Japanese retrospective analysis in pediatric AML with *RUNX1*‐*RUNX1 T1* identified CD19 negativity as the sole significant risk factor for relapse, a result still true even when restricting patients without the *KIT* exon 17 mutation, implying a biological difference between CD19‐positive and CD19‐negative AML patients with *RUNX1*‐*RUNX1 T1*.^20^ We speculate that an absence of surface antigen CD19 on t(8;21) leukemic cells may escape the T cell‐mediated immunologic clearance mechanism, leading to the persistence of minimal residual disease and eventually leukemia relapse, which is needed to be confirmed by further researches. Combination of CD19 and *KIT* can further optimize the relapse risk stratification of t(8;21) AML, which enables us to identify a small subset of patients with both *KIT*mut and CD19(−) as having the highest relapse risk.

So far, our report encompassed relatively larger sample size to generate information of clinicopathological and NGS data in multivariate analysis. The main limitations of this study rested with its biased allocation of patients, incompleteness of immunophenotypic records, and loss to follow‐up after referral, which might impair the power of statistical tests. Also, there were censoring heterogeneities resulting from donor availability and timing of transplantation for those undergoing allo‐SCT. Prospectively randomized trials expanding patient subjects and prolonging follow‐up time are warranted to validate the clinical significance of these prognostic factors in t(8;21) AML subpopulations.

Taken together, we conclude that *KIT*mut, predominantly D816, adversely affects the remission of t(8;21) AML patients induced with standard‐dose Ara‐C, which can be abrogated by dose‐intensified Ara‐C containing regimen. Negative CD19 predicts increased relapse risk independently of *KIT*mut and Ara‐C induction intensity. The distinct *KIT*‐D816, rather than general *KIT*mut, may be incorporated into the prognostication system of t(8;21) AML. Mutual combination of CD19 and *KIT*mut can deliver a more definite risk stratification profile for t(8;21) AML, thereby guiding an individually risk‐adapted curative treatment, such as allo‐SCT.

## CONFLICT OF INTEREST

The authors declare that they have no conflict of interests.

## Supporting information

Table S1

Table S2

Table S3

## Data Availability

The datasets used and/or analyzed during the current study are available from the corresponding author on reasonable request.
