# Supplementary material for: Role of CD19 and specific KIT‐D816 on risk stratification refinement in t(8;21) acute myeloid leukemia induced with different cytarabine intensities
Source: Cancer Med. 2020 Dec 31;10(3):1091–102. doi: 10.1002/cam4.3705 (PMC7897948; doi:10.1002/cam4.3705)
Supplement: Supplementary file 1 — Table S1 [file CAM4-10-1091-s001.docx]

**Supplementary table S1.** Baseline clinical features among t(8;21) AML patients grouped by induction

| Median (range) or n (%) | | Whole | SD Ara-C | ID Ara-C | *P*# |
| --- | --- | --- | --- | --- | --- |
| Number of cases | | 207 | 107 | 90 | NA |
| Age, years | | 35 (16-71) | 36 (16-67) | 31 (16-54) | 0.064 |
| Age < 35 | | 108 (52.2) | 50 (46.7) | 57 (63.3) | ***0.020*** |
| Age ≥ 35 | | 99 (47.8) | 57 (53.3) | 33 (36.7) |  |
| Sex, male:female | | 109:98 | 57:50 | 49:41 | 0.869 |
| WBC count, ×10^9^/L | | 8.7 (0.9-155.0) | 8.7 (1.3-155.0) | 8.7 (0.9-129.0) | 0.616 |
| WBC < 10 | | 111 (53.6) | 58 (54.2) | 48 (53.3) | 0.903 |
| WBC ≥ 10 | | 96 (46.4) | 49 (45.8) | 42 (46.7) |  |
| Hb level, g/L | | 76 (39-145) | 74 (39-145) | 81 (39-121) | 0.070 |
| Hb < 80 | | 116 (56.0) | 68 (63.6) | 42 (46.7) | ***0.017*** |
| Hb ≥ 80 | | 91 (44.0) | 39 (36.4) | 48 (53.3) |  |
| PLT count, ×10^9^/L | | 30 (2-202) | 28 (2-195) | 32 (6-202) | 0.081 |
| PLT < 30 | | 102 (49.3) | 57 (53.3) | 39 (43.3) | 0.165 |
| PLT ≥ 30 | | 105 (50.7) | 50 (46.7) | 51 (56.7) |  |
| *RUNX1-RUNX1T1* level, copies/*ABL* copies | | 158.9 (18.9-817.6) | 179.4 (28.1-675.0) | 152.2 (18.9-817.6) | 0.109 |
| < 150 | | 72/160 (45.0) | 28/70 (40.0) | 42/85 (49.4) | 0.241 |
| ≥ 150 | | 88/160 (55.0) | 42/70 (60.0) | 43/85 (50.6) |  |
| *WT1* level, copies/*ABL* copies | | 30.8 (0.2-312.7) | 22.7 (0.2-312.7) | 39.0 (0.3-182.4) | 0.085 |
| *WT1* < 30 | | 64/129 (49.6) | 31/57 (54.4) | 30/66 (45.5) | 0.323 |
| *WT1* ≥ 30 | | 65/129 (50.4) | 26/57 (45.6) | 36/66 (54.5) |  |
| Immunophenotype, n/N (%)* | |  |  |  |  |
|  | CD34 | 202/205 (98.5) | 105/105 (100.0) | 88/90 (97.8) | 0.212F |
|  | TdT | 52/201 (25.9) | 24/101 (23.8) | 27/90 (30.0) | 0.331 |
|  | HLA-DR | 200/205 (97.6) | 104/105 (99.0) | 86/90 (95.6) | 0.279C |
|  | CD117 | 204/205 (99.5) | 105/105 (100.0) | 89/90 (98.9) | 0.462F |
|  | CD13 | 195/205 (95.1) | 101/105 (96.2) | 84/90 (93.3) | 0.565C |
|  | CD33 | 200/204 (98.0) | 103/105 (98.1) | 87/89 (97.8) | 1.000C |
|  | CD123 | 192/196 (98.0) | 100/101 (99.0) | 84/86 (97.7) | 0.888C |
|  | CD38 | 191/199 (96.0) | 101/103 (98.1) | 81/86 (94.2) | 0.309C |
|  | CD64 | 10/178 (5.6) | 6/90 (6.7) | 2/80 (2.5) | 0.359C |
|  | CD56 | 157/188 (83.5) | 81/96 (84.4) | 69/83 (83.1) | 0.822 |
|  | MPO | 194/199 (97.5) | 100/101 (99.0) | 85/89 (95.5) | 0.239C |
|  | CD9 | 58/133 (43.6) | 30/68 (44.1) | 25/59 (42.4) | 0.843 |
|  | CD19 | 154/204 (75.5) | 79/105 (75.2) | 69/89 (77.5) | 0.709 |
|  | CD79a | 43/201 (21.4) | 22/101 (21.8) | 17/90 (18.9) | 0.620 |
|  | CD7 | 22/189 (11.6) | 13/96 (13.5) | 5/84 (6.0) | 0.090 |
| Cytogenetics | |  |  |  |  |
|  | Translocation alone | 74 (35.7) | 39 (36.4) | 32 (35.6) | 0.897 |
|  | Additional LOS | 102 (49.3) | 52 (48.6) | 47 (52.2) | 0.612 |
|  | Additional del(9q) | 12 (5.8) | 3 (2.8) | 7 (7.8) | 0.208C |
|  | Additional ≥ 2 other abnormalities | 30 (14.5) | 12 (11.2) | 15 (16.7) | 0.268 |
|  | Additional ≥ 3 other abnormalities | 14 (6.8) | 8 (7.5) | 3 (3.3) | 0.207 |
|  | Three-way translocations | 13 (6.3) | 7 (6.5) | 6 (6.7) | 0.972 |

[**Abbreviation**](http://dict.cn/abbreviation)**s and Annotations:**

SD, standard-dose; ID, intermediate-dose; Ara-C, cytarabine; WBC, white blood cell; Hb, hemoglobin; PLT, platelet; LOS, loss of sex chromosome; F, Fisher' exact test; C, continuity correction; NA, not applicable; n/N (%)*, percentage according to available data; *P*#, the significance were obtained between SD and ID Ara-C group from Chi-square test after crosstabulation for categorical variables (Pearson results unless otherwise specified) or from Mann-Whitney U test for continuous variables of non-normal distribution. Parameters showing statistical significance are highlighted in bold and italic.
