# Supplementary material for: Role of CD19 and specific KIT‐D816 on risk stratification refinement in t(8;21) acute myeloid leukemia induced with different cytarabine intensities
Source: Cancer Med. 2020 Dec 31;10(3):1091–102. doi: 10.1002/cam4.3705 (PMC7897948; doi:10.1002/cam4.3705)
Supplement: Supplementary file 2 — Table S2 [file CAM4-10-1091-s002.docx]

**Supplementary table S2.** Genetic characteristics among t(8;21) AML patients grouped by induction

| Mutations | Whole | SD Ara-C | ID Ara-C | *P*# |
| --- | --- | --- | --- | --- |
| Number of cases, n (%) | 186 | 95 | 85 | NA |
| Signaling pathways |  |  |  |  |
| *KIT* | 85 (45.7) | 47 (49.5) | 35 (38.7) | 0.264 |
| *KIT*-D816 | 42 (22.6) | 29 (30.5) | 11 (12.9) | ***0.005*** |
| *KIT*-N822 | 39 (21.0) | 17 (17.9) | 21 (24.7) | 0.264 |
| *NRAS* | 28 (15.1) | 14 (14.7) | 14 (16.5) | 0.749 |
| *FLT3* | 24 (12.9) | 12 (12.6) | 12 (14.1) | 0.770 |
| *FLT3*-ITD | 12 (6.5) | 7 (7.4) | 5 (5.9) | 0.690 |
| *FLT3* others | 12 (6.5) | 5 (5.3) | 7 (8.2) | 0.425 |
| *CSF3R* | 19 (10.2) | 8 (8.4) | 10 (11.8) | 0.455 |
| *RELN* | 17 (9.1) | 8 (8.4) | 9 (10.6) | 0.620 |
| *JAK2* | 16 (8.6) | 8 (8.4) | 8 (9.4) | 0.816 |
| *NOTCH1* | 13 (7.0) | 10 (10.5) | 3 (3.5) | 0.070 |
| *SH2B3* | 10 (5.4) | 8 (8.4) | 2 (2.4) | 0.147C |
| Epigenetic regulators |  |  |  |  |
| *KMT2D* | 20 (10.8) | 10 (10.5) | 9 (10.6) | 0.989 |
| *TET2* | 17 (9.1) | 8 (8.4) | 9 (10.6) | 0.620 |
| *ASXL1* | 15 (8.1) | 9 (9.5) | 6 (7.1) | 0.558 |
| *EP300* | 11 (5.9) | 6 (6.3) | 5 (5.9) | 0.904 |
| *CREBBP* | 10 (5.4) | 6 (6.3) | 4 (4.7) | 0.885 |
| Tumor suppressors |  |  |  |  |
| *FAT1* | 28 (15.1) | 14 (14.7) | 13 (15.3) | 0.917 |
| *ATM* | 11 (5.9) | 4 (4.2) | 7 (8.2) | 0.260 |
| Number of mutations | 3 (0-11) | 3 (0-11) | 4 (0-8) | 0.111 |

[**Abbreviation**](http://dict.cn/abbreviation)**s and Annotations:**

SD, standard-dose; ID, intermediate-dose; Ara-C, cytarabine; NA, not applicable; *P*#, the significance were obtained between SD and ID Ara-C group from Chi-square test after crosstabulation for categorical variables (Pearson results unless otherwise specified). Parameters showing statistical significance are highlighted in bold and italic.
