# Supplementary material for: Role of CD19 and specific KIT‐D816 on risk stratification refinement in t(8;21) acute myeloid leukemia induced with different cytarabine intensities
Source: Cancer Med. 2020 Dec 31;10(3):1091–102. doi: 10.1002/cam4.3705 (PMC7897948; doi:10.1002/cam4.3705)
Supplement: Supplementary file 3 — Table S3 [file CAM4-10-1091-s003.docx]

**Supplementary table S3.** Screened univariate Chi-square results of factors impacting the CR rate among t(8;21) AML patients grouped by induction

| Factors | | Entire cohort, n=197 | |  | SD Ara-C, n=107 | |  | ID Ara-C, n=90 | | *P*# |
| --- | --- | --- | --- | --- | --- | --- | --- | --- | --- | --- |
|  |  | **CR rate, n/N (%)** | ***P**** |  | **CR rate, n/N (%)** | ***P**** |  | **CR rate, n/N (%)** | ***P**** |  |
| WBC count | < 10 | 96/104 (92.3) | ***0.033*** |  | 49/57 (86.0) | 0.058 |  | 47/47 (100.0) | 0.220F | ***0.021C*** |
|  | ≥ 10 | 74/90 (82.2) |  |  | 34/48 (70.8) |  |  | 40/42 (95.2) |  | ***0.003*** |
| CD19 | (−) | 36/44 (81.8) | 0.136 |  | 17/25 (68.0) | 0.453 |  | 19/19 (100.0) | 1.000F | ***0.020C*** |
|  | (+) | 131/147 (89.1) |  |  | 64/78 (82.1) |  |  | 67/69 (97.1) |  | ***0.003*** |
| *KIT* | (−) | 92/98 (93.9) | ***0.005*** |  | 42/48 (87.5) | ***0.029*** |  | 50/50 (100.0) | 0.167F | ***0.031C*** |
|  | (+) | 64/80 (80.0) |  |  | 31/45 (68.9) |  |  | 33/35 (94.3) |  | ***0.005*** |
| *KIT*-D816 | (−) | 128/140 (91.4) | ***0.008C*** |  | 56/66 (84.8) | ***0.020*** |  | 72/74 (97.3) | 1.000F | ***0.009*** |
|  | (+) | 28/38 (73.7) |  |  | 17/27 (63.0) |  |  | 11/11 (100.0) |  | 0.052C |
| Entire cohort | | 170/194 (87.6) | NA |  | 83/105 (79.0) | NA |  | 87/89 (97.8) | NA | ***<0.001*** |

[**Abbreviation**](http://dict.cn/abbreviation)**s and Annotations:**

SD, standard-dose; ID, intermediate-dose; Ara-C, cytarabine; WBC, white blood cell; F, Fisher' exact test; C, continuity correction; NA, not applicable; *P**, the significance between factors of different levels; *P*#, the significance between SD and ID Ara-C group; Prior to multivariate Logistic analysis, the univariate Chi-square test preliminarily screens predictors impacting the CR rate in entire cohort or in both induction groups. Comparisons are performed between different layers of these factors. By default, All *P* values are from Pearson Chi-square results, otherwise denoted with “C” (continuity correction) or “F” (Fisher' exact test), where appropriate. Parameters showing statistical significance are highlighted in bold and italic.
